# Hydrology and the future of the Greenland Ice Sheet

**DOI:** 10.1038/s41467-018-05002-0

**Published:** 2018-07-16

**Authors:** Gwenn E. Flowers

**Affiliations:** Department of Earth Sciences, Simon Fraser University, 8888 University Drive, Burnaby, BC V5A 1S6 Canada

**Keywords:** Climate change, Cryospheric science, Hydrology

## Abstract

Detection, attribution and projection of mass loss from the Greenland Ice Sheet has been a central focus of the glaciological community, with surface meltwater thought to play a key role in feedbacks that could accelerate sea-level rise. While the prospect of runaway sliding has faded, much remains uncertain when it comes to the role of surface runoff and subglacial discharge in Greenland’s future.

With Greenland’s reservoir of ice exceeding 7 m of sea-level equivalent (SLE), its accelerating mass loss regularly captures headlines. About 60% of Greenland’s 1991–2015 mass loss (~0.47 ± 0.23 mm per year SLE) has been attributed to surface mass balance^1^, the net difference between snowfall and melt. The remaining 40% is attributed to uncompensated ice-flow into the ocean, or dynamic mass loss. Observations in the early 2000s linked seasonal meltwater production to ice-sheet acceleration, disrupting the prevailing view of the Greenland Ice Sheet as unresponsive to surface melt. This intuitive link based on water reducing friction at the ice–bed interface, familiar from decades of work on alpine glaciers, led to proclamations of a looming positive feedback between surface melt and Greenland’s dynamic mass loss. Fifteen years of intensive research (Fig. 1) now offers a more nuanced view^2^ that allows us to reconsider the question: What role will surface melt—which stands to increase in lock step with atmospheric warming—play in Greenland’s future?

**Fig. 1:**
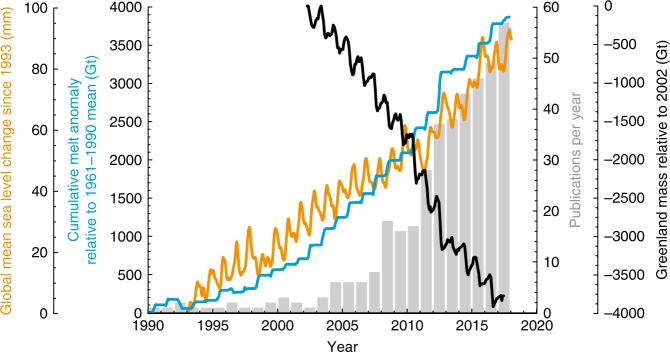
Greenland melt and mass loss, global sea level and related research. Published research on Greenland Ice Sheet hydrology and dynamics, broadly defined, has increased sharply this century (grey bars), against a backdrop of increasing surface melt (blue), ongoing ice-sheet mass loss (black) and rising global sea levels (orange). Publications from Web of Science with manual additions/deletions. Ice-sheet-wide melt anomalies from RACMO2.3p2, statistically downscaled from 5.5 to 1 km (unpublished data from B.P.Y. Noël and M.R. van den Broeke, Institute for Marine and Atmospheric Research, Utrecht University). Mass loss from Jet Propulsion Laboratory under the auspices of the NASA MEaSUREs program. Uses GRACE data from JPL RL05M.1 Mascon Solution: Version 2 (Wiese, D. N., D.-N. Yuan, C. Boening, F. W. Landerer, and M. M. Watkins (2016) JPL GRACE Mascon Ocean, Ice, and Hydrology Equivalent HDR Water Height RL05M.1 CRI Filtered Version 2., Ver. 2., PO.DAAC, CA, USA. Dataset accessed [2018-05-20] at 10.5067/TEMSC-2LCR5). Sea-level data from NASA Goddard Space Flight Center under the auspices of the NASA MEaSUREs program (GSFC. 2017. Data are smoothed (60-day Gaussian type filter) with respect to 20-year mean and have GIA applied. Global Mean Sea Level Trend from Integrated Multi-Mission Ocean Altimeters TOPEX/Poseidon, Jason-1, OSTM/Jason-2 Version 4.2 Ver. 4.2 PO.DAAC, CA, USA. Dataset accessed [2018-05-20] at 10.5067/GMSLM-TJ142)

## Water and heat flow in the near surface

With most of the Greenland Ice Sheet blanketed in snow and firn (a transitional material between snow and ice), and melting conditions reaching further inland as observed during the extraordinary summer of 2012, near-surface hydrology stands to play an increasingly important role in connecting surface melt and sea-level rise. The discovery of vast stores of liquid water in the porous near-surface firn^3^ highlights a potential feedback termed cryo-hydrologic warming^4^, whereby latent heat released by refreezing meltwater warms the surrounding ice, accelerating its deformation. There is some observational evidence for this mechanism, for example, in anomalously warm ice found in western Greenland. However, the effect of cryo-hydrologic warming is expected to be limited at least on centennial timescales, with the Greenland Ice Sheet being more sensitive to climate forcing and bed conditions than internal thermal regime^5^. The more significant effect of refreezing may be its role in limiting the porosity and permeability of the firn^6^. Combined with reduced firn extent, various densification processes and diminished cold content, the firn aquifer’s future buffering capacity of Greenland’s contribution to sea level stands to be compromised. The firn aquifer may also serve to concentrate water supplies in a manner conducive to hydrofracture, allowing water stored in the near-surface to reach the base of the ice-sheet.

## From surface to bed through 1000 m of cold ice

Below the firn line, kilometre-scale sapphire-blue lakes, fed by networks of surface streams, dot the flanks of the Greenland Ice Sheet in summer. Their disappearance is one of the most striking features in time-lapse satellite imagery, prompting questions about their role in delivering water to the ice-sheet interior. Some of these lakes drain by overtopping into downstream crevasses and moulins (vertical shafts in the ice created by flowing water). Others drain rapidly through in-situ fractures that may propagate all the way to the bed, in some cases associated with uplift and/or sliding of the ice sheet caused by distal drainage events^7^. Water impinging on the bed in this way has a short-lived effect on local ice flow^8^ that may or may not be significant when the cumulative impacts of numerous lakes are considered, but does facilitate the formation of longer-lived moulins. Seasonal cycles of surface meltwater supply compete with the evolution of subglacial drainage-system efficiency, with ice-flow speeds seemingly more dependent on meltwater variability than volume^9^. Even negative feedbacks between surface melt and annual ice motion have been documented in multi-year studies^10^, particularly at lower elevations where a well-developed drainage system permits efficient evacuation of water.

Just as the melting margins of Greenland were beginning to seem like an outsized alpine glacier with its seasonal see-saw between efficient and inefficient subglacial drainage, borehole studies highlighted limits to the inland extent of an efficient drainage system^11^ and the importance of hydraulically isolated regions of the bed^12^. While persistent surface-to-bed connections appear to have little influence on long-term ice flux, the wider implications of supraglacial lake drainage remain murky. A Greenland-wide assessment revealed a regionally differentiated response, with fewer drainage events in areas experiencing the greatest dynamic mass losses^13^. Though lakes are projected to form further inland with rising temperatures and poised to deliver water to increasingly sensitive areas of the bed^14^, whether lake drainage will be effective at these inland sites is unclear.

## Subglacial discharge and Greenland’s marine margins

Most work investigating the direct impacts of surface meltwater has targeted land-terminating outlet glaciers. Marine-terminating glaciers are fundamentally different however, in that ice–ocean interactions permit highly non-linear and unstable behaviour with profound impacts on glacier retreat and the attendant export of ice to the ocean. Sediment-laden freshwater plumes can be seen rising from subglacial conduits to the fjord surface, implicating glacier melt in fjord circulation dynamics. These meltwater inputs draw warm ocean water at depth toward the ice front, where sub-marine meltrates can reach metres per day^15^. Complexities arise, however, from the particulars of fjord configuration, ice depth, subglacial discharge rates, the geometry of subglacial conduits and externally forced ocean currents and fjord stratification^16,17^.

Though it is too early to generalize on the importance of ice-sheet hydrology for processes at Greenland’s marine margins, current indications are that surface melt does have an important role in driving dynamics at the ice–ocean interface where marine-terminating glaciers are most sensitive. Although the contributions to Greenland’s dynamic mass loss since 2000 are concentrated in relatively few outlet glaciers, there may be other glaciers with geometries and fjords primed for retreat. In contrast to Antarctica, most of Greenland’s marine-terminating glaciers are ultimately destined to retreat onto dry land, spelling a less dynamic future ice sheet. In the meantime, even a decade of perturbation at the ice front can lead to a century of ice-sheet drawdown and committed sea-level rise^18^, so the effects of dynamic thinning will be with us well into the future.

## Current challenges and priorities for future research

While technological advances in observing systems allow unprecedented monitoring, there are fundamental questions that remain. How is surface meltwater partitioned between infiltration and runoff^19^? How rapidly will this partitioning change as a function of snow/firn extent, porosity and permeability^20^? Where does this water access the bed^21^? How pervasive and persistent might efficient subglacial drainage become^11^, and what role do the bed areas isolated from the drainage system play in ice-sheet dynamics^12^? How important is ice-sheet hydrology in driving mass loss from marine-terminating outlets, both at the ice front and upstream^22^?

Satellite remote sensing is now able to provide regular ice-sheet-wide snapshots of ice-surface velocities and net mass changes (e.g. NASA MEaSUREs program), while ground-based and airborne geophysical data (e.g. through Operation IceBridge) also play an important role in spatially extended studies. But many hydrological variables remain challenging to observe, let alone predict. Supraglacial channels of several metres width are visible from space, but depths and water fluxes are difficult to obtain. Discrete injection of meltwater through moulins is a key feature of the drainage system, but predicting moulin formation can be challenging. The englacial and subglacial drainage systems are almost entirely obscured from view, directly observable only at the point-scale through labour-intensive borehole drilling or natural portals such as moulins. Models of ice-sheet hydrology, still in their infancy, tend to resolve individual conduit elements, making efficient coupling with large-scale ice-flow models still a thing of the future. They also require parameterization of metre-scale bed roughness and higher resolution bed topography than is widely available.

Continued progress will require sustained efforts to better image the ice-sheet interior and bed as well as coastal bathymetry, combined with data assimilation exercises such as BedMachine designed to produce seamless and self-consistent public datasets^23^. Information on bed composition^24^ and roughness at multiple scales would help reduce unnecessary ambiguity for models. From surface to bed to ice front, we also need to exercise creativity in upscaling process-based studies, and vigilance in the unavoidable geographical biases imposed by field logistics. Productive partnerships between disciplinary specialists will move the science forward fastest, as demonstrated in grassroots initiatives like GRISO (https://web.whoi.edu/griso/).

As a community, we have developed a nuanced understanding of the impact of surface meltwater on the dynamics of the Greenland Ice Sheet, but ice sheets are more than just their net contributions to global sea level. They sculpt landscapes, deliver sediment, solutes and nutrients to the ocean, gravitationally shape our coastlines and influence global climate. Although the threat of surface meltwater to Greenland’s stability does not appear as grave as it once did, the details of how and where meltwater is stored, how it is ultimately transported through the ice sheet to the ocean, and what physical, chemical and biological effects it has along the way should be priorities for future research. There we have barely scratched the surface.
