# Author Correction: Hydrology and the future of the Greenland Ice Sheet

**DOI:** 10.1038/s41467-019-11573-3

**Published:** 2019-07-31

**Authors:** Gwenn E. Flowers

**Affiliations:** Department of Earth Sciences, Simon Fraser University, 8888 University Drive, Burnaby, BC V5A 1S6 Canada

**Keywords:** Climate change, Cryospheric science, Hydrology

Correction to: *Nature Communications* 10.1038/s41467-018-05002-0, published online 16 July 2018.

The original version of this Article contained an error in the final sentence of the legend to Fig. 1, which incorrectly read ‘Dataset accessed [2018-05-20] at 10.5067/GMSLM-TJ42’. The correct version states ‘10.5067/GMSLM-TJ142’ in place of ‘10.5067/GMSLM-TJ42’. This has been corrected in both the PDF and HTML versions of the Article.

